# Natural Urease Inhibitors Reduce the Severity of Disease Symptoms, Dependent on the Lifestyle of the Pathogens

**DOI:** 10.3390/jof9070708

**Published:** 2023-06-28

**Authors:** Lala Aliyeva-Schnorr, Carola Schuster, Holger B. Deising

**Affiliations:** 1 Chair for Phytopathology and Plant Protection, Institute for Agricultural and Nutritional Sciences, Faculty of Natural Sciences III, Martin-Luther-University Halle-Wittenberg, Betty-Heimann-Str. 3, D-06120 Halle (Saale), Germany; 2 SKW Stickstoffwerke Piesteritz GmbH, Möllensdorfer Str. 13, D-06886 Lutherstadt Wittenberg, Germany

**Keywords:** biotrophic fungi, *Colletotrichum graminicola*, necrotrophic fungi, plant protection, urease inhibitor

## Abstract

The development of new anti-ureolytic compounds is of great interest due to the newly discovered role of urease inhibitors in crop protection. Purine degradation and the generation of ammonium by urease are required for the full virulence of biotrophic and hemibiotrophic fungal plant pathogens. Accordingly, chemicals displaying urease inhibitor activity may be used as a novel class of fungicides. Several urease inhibitors belonging to different chemical classes are known, and some compounds have been developed as urea fertilizer additives. We tested whether the natural urease inhibitors *p*-benzoquinone (*p*-HQ) and hydroquinone (HQ), as well as the synthetic inhibitors isopropoxy carbonyl phosphoric acid amide (iCPAA), benzyloxy carbonyl phosphoric acid amide (bCPAA), and dipropyl-hexamino-1,3 diphosphazenium chloride (DDC), prevent or delay plant infection caused by pathogens differing in lifestyles and host plants. *p*-BQ, HQ, and DCC not only protected maize from infection by the hemibiotroph *C. graminicola*, but also inhibited the infection process of biotrophs such as the wheat powdery mildew fungus *Blumeria graminis* f. sp. *tritici* and the broad bean rust fungus *Uromyces viciae-fabae*. Interestingly, the natural quinone-based compounds even reduced the symptom severity of the necrotrophic fungi, i.e., the grey mold pathogen *B. cinerea* and the Southern Leaf Spot fungus *C. heterostrophus*, to some extent. The urease inhibitors *p*-BQ, HQ, and DCC interfered with appressorial penetration and confirmed the appropriateness of urease inhibitors as novel fungicidal agents.

## 1. Introduction

Urease has been identified as an important virulence factor in several mammalian pathogens, including dimorphic fungi such as the ascomycete *Coccioides posadasii* and the basidiomycete *Cryptococcus neoformans*, and in bacteria like *Helicobacter pylori*, *Proteus mirabilis*, and others [1]. Great interest in the role of urease in pathogenesis was sparked by the discovery that the bacterium *H. pylori* is associated with the occurrence of gastritis and gastric cancer. *H. pylori* grows well at pH values of 6 to 8 but does not survive at pH values below 4 [2]. The acidic environment of the human stomach acidifies the bacterial periplasm and, consequently, the proton-gated urea channel UreI imports urea into the bacterial cytosol. Here, the enzyme urease hydrolyses urea to ammonia and bicarbonate to buffer protons in the periplasm [3,4]. Moreover, apart from the role of urease in pH adjustment in the bacterial micro-environment, its reaction product ammonia is highly toxic, resulting in epithelial cell damage at the sites of *H. pylori* infection. The role of the urease in pH adjustment and tissue damage was not only found in *H. pylori*. Studies employing the human pathogenic fungi *C. posadasii* and *C. neoformans* suggested a conserved role of urease in bacterial and fungal pathogenesis. In these pathogens, ammonia-mediated cell damage is required for lung invasion, inhibition of phagocyte function, and for accessing the bloodstream. After getting trapped in small capillaries, urease-generated ammonia again causes cell damage and allows the fungus to grow from the capillary, propagate, and cause severe damage in the host tissue [5,6]. Accordingly, urease-deficient strains of *C. neoformans* were significantly reduced in virulence, and higher concentrations of antigen-presenting cells were found in the brains of mice inoculated with the urease-positive strain compared with the control animals infected with urease-negative strains [5,7,8]. However, dissemination patterns in the brain differed markedly after intravenous inoculation, strongly suggesting a role for urease in facilitating blood-brain invasion in this fungus [9]. For further details on the role of urease in bacterial and fungal infections of humans, the reader is referred to an excellent review by Rutherford [5].

While the role of urease in human pathogens can be considered well-understood, little is known about the virulence function of this enzyme in plant pathogens. *Agrobacterium tumefaciens*-mediated random mutagenesis experiments have identified the allantoicase gene of the maize anthracnose fungus *Colletotrichum graminicola* as a virulence factor [10] and suggested that the generation of urea and, subsequently, ammonium via the purine degradation pathway may be a prerequisite for full virulence. Indeed, urease-deficient mutants of this fungus showed virulence defects and supplementing the infection droplets with ammonium restored the virulence of the Δ*ure1* deletion strains [11]. Moreover, the addition of the commercially available urease inhibitor acetohydroxamic acid (AHA), which is structurally related to urea but not hydrolysable by urease [10], reduced the virulence of the *C. graminicola* WT strain and strengthened the idea that chemical inhibitors of urease activity may represent a novel class of agents protecting plants from fungal disease [11].

The plant surface is extremely low in nitrogen [12,13], and purine degradation and urease-mediated ammonium formation are therefore thought to be of particular importance at the pre-penetration stages of plant infection. However, this idea may only hold true for biotrophic and hemibiotrophic pathogens, which get access to nitrogen in the host tissue only after establishing a stable parasitic interaction with the host plant, as indicated by the differentiation of haustoria or comparable cells specialized in nutrient uptake [14,15]. Necrotrophs, by contrast, secrete toxins prior to plant invasion, kill the host epidermal cell, and may gain access to nitrogenous metabolites even before host invasion [16]. Thus, it is unknown whether urease inhibitors are also effective against necrotrophic pathogens.

The role of urease in pH increases in plant-infecting pathogens may also be considered. The broad bean rust fungus *Uromyces viciae-fabae* secretes the plant cell wall-degrading enzyme polygalacturonate lyase when haustorial mother cells are differentiated in the host, i.e., immediately at the site of host cell invasion. This enzyme has an extremely alkaline pH optimum of higher than 10 [17], and increasing the pH at the infection site may be required to activate this pectic enzyme. Comparably, cell wall-degrading enzymes supporting appressorial penetration may require a urease-mediated pH increase for host invasion.

Urease inhibitors belonging to several chemical groups [18] are widely used to increase the efficacy of urea fertilization in agricultural soils. We thus wondered whether compounds used in soil application may also be suited to inhibit pathogens employing distinct lifestyles and support plant health when applied to above-ground plant organs. Therefore, we tested the specificity of natural and synthetic urease inhibitors differing in their mode of action. The results of this study may expand the portfolio of fungicidal chemistries to be used in plant protection.

## 2. Materials and Methods

### 2.1. Fungal and Plant Material and Infection Experiments

Infection experiments were performed on leaves of maize (*Zea maize* cv. Mikado; KWS SAAT SE, Einbeck, Germany), winter wheat (*Triticum aestivum* cv. Winnetou; Saatzucht Firlbeck GmbH & Co. KG, Atting, Germany), broad bean (*Vicia faba* cv. Fuego; Norddeutsche Pflanzenzucht Hans-Georg Lembke KG, Holtsee, Germany), and on strawberry (*Fragaria x ananassa* cv. Ostara) fruits. Experiments were performed in growth chambers (AR-75L, Persival Scientific Inc., Perry, IA 50220, USA) at 22 °C. Light intensities were ~140 μmol/m^2^/s, except for *Z. mays*, which was grown at ~300 μmol/m^2^/s at 25 °C). Strains and infection experiments for *Blumeria graminis* f. sp. *Tritici*, *Uromyces viciae-fabae*, and *Colletotrichum graminicola* were as described [7]. The wheat powdery mildew fungus *B. graminis* f. sp. *tritici* is a field single-spore-isolate (BgtEV01) collected at the Experimental Station of Martin Luther University, and maintained on susceptible *T. aestivum* cv. Winnetou plants were grown in a greenhouse at 21 ± 1 °C. The broad bean rust fungus, *U. viciae-fabae*, isolate I2, was provided by Kurt Mendgen, University of Konstanz, Germany. The wild-type strain CgM2 (syn. M.1.001) of *C. graminicola* was from the strain collection of Ralph L. Nicholson, Purdue University, IN, USA, and maintained as described [19]. The Southern Leaf Spot fungus *Cochliobolus heterostrophus* (lab strain C12) was provided by Stefan Wirsel (MLU Halle, Halle, Germany) and the grey mold fungus *Botrytis cinerea* was from Paul Tudzynski (Westfälische Wilhelms-Universität Münster, Münster, Germany). *C. heterostrophus* and *B. cinerea* were kept on Potato Dextrose Agar (PDA; BD DIFCO™, Franklin Lakes, NJ, USA).

For urease inhibitor treatment, spore suspensions of *C. graminicola*, *U. viciae-fabae*, *C. heterstrophus*, and *B. cinerea* were adjusted to contain 10 mM acetohydroxamic acid (AHA; Sigma-Aldrich, Darmstadt, Germany) [7], or 1, 5, or 10 mM of the urease inhibitors *p*-benzoquinone (*p*-BQ), hydroquinone (HQ), isopropoxy carbonyl phosphoric acid amide (iCPAA), benzyloxy carbonyl phosphoric acid amide (bCPAA), and dipropyl-hexamino-1,3-diphosphacenium chloride (DDC). To test the effect of urease inhibitors on powdery mildew infection, 1, 5, or 10 mM of the inhibitors were sprayed onto leaves until run-off and allowed to dry for 2 h prior to inoculation. To analyze the effect of the compounds on rust infection structure differentiation in vitro, urediniospores of *U. viciae-fabae* were suspended in 0.02% (*v*/*v*) Tween 20. The inoculum was adjusted to contain 1, 5, or 10 mM of the inhibitors and sprayed onto thigmo-inductive polyethylene sheets [20]. At 24 h post-inoculation (hpi), infection structures were stained with aniline blue (Sigma-Aldrich, Darmstadt, Germany) and counted. To analyze the effect of urease inhibitors on disease symptom severity caused by the hemibiotroph *C. graminicola*, detached maize leaves were inoculated with 10 µL droplets of a suspension containing 10^6^ conidia/mL 0.02% (*v*/*v*) Tween 20 and incubated at 23 °C in darkness. The development of disease symptoms was scored and photographed daily. To analyze appressorial penetration rates by microscopy, leaves were inoculated with 10 µL droplets of a suspension containing 10^5^ conidia/mL 0.02% (*v*/*v*) Tween 20, and penetration rates were counted at 48 hpi [21]. For *B. cinerea* infections, strawberry fruits were surface sterilized by incubation in 70% ethanol for 3 min, followed by three washes in sterile water. Spray inoculation with *B. cinerea* to the point of run-off was performed with 10^4^ spores/mL 0.02% (*v*/*v*) Tween 20. To analyze the effect of urease inhibitors, *p*-HQ, HQ, iCPAA, bCPAA, and DDC were added to the conidial suspensions at a concentration of 10 mM. Inoculated strawberries were kept in a climate chamber at 23 °C, 50% relative humidity, and a 12 h photoperiod. For *C. heterostrophus* infections, 10 µL of a spore suspension (10^4^ spores per ml of 10 mM urease inhibitors in 0.02% (*v*/*v*) Tween 20) were droplet-inoculated onto detached leaf segments as described for *C. graminicola*. For phytotoxicity tests, 10 mM solutions of each compound were sprayed onto leaf segments or fruits. Plants were incubated as described above, and the formation of chloroses or necroses was checked daily until 5 days post-treatment (dpt).

### 2.2. Inhibition of Urease Activity

To test the efficiency of the inhibitors, in vitro activity assays were performed using Jackbean (*Canavalia ensiformis*) urease (EC 3.5.1.5, Sigma Aldrich, Taufkirchen, Germany) dissolved in 0.1 M K-phosphate buffer, pH 7.3. To quantify urease activity, liberation of ammonium from urea was determined spectrophotometrically at λ620 nm, using the Berthelot method in a two-step reaction with hypochlorite and salicylate [22]. The reaction mixture contained 50 mM urea; 0.1 M K-phosphate buffer, pH 7.3; and 30 U of jackbean urease in a total volume of 1.0 mL. After incubation for 30 min at 37 °C, 0.5 mL of sodium hypochlorite (0.5% (*v*/*v*) free chlorine) in 0.5 M NaOH and 0.1 M salicylic acid in 1.0 M NaOH were added, and color development was measured after 15 min of incubation at room temperature. The urease inhibitors were used at concentrations of 1, 5, or 10 mM.

### 2.3. Microscopy

Brightfield and differential interference contrast (DIC) microscopy was performed with a Nikon Eclipse 600 microscope (Nikon, Düsseldorf, Germany). Digital images were taken using a DS-5M camera (Nikon, Düsseldorf, Germany). Image processing was done with the software package Lucia 4.61 (Nikon GmbH, Düsseldorf, Germany). Image analysis was performed using ImageJ [23].

### 2.4. Statistical Analysis

All plant infection assays were performed as three independent replicates. Urease activity assays also represent data from three independent measurements. Statistical significance was analyzed by applying the Tukey post hoc test with one-way analysis of variance at either *p* ≤ 0.01 for the plant infection assays or *p* ≤ 0.05 for the urease activity test. Significant differences were indicated by asterisks in the results.

## 3. Results

**Figure 1:**
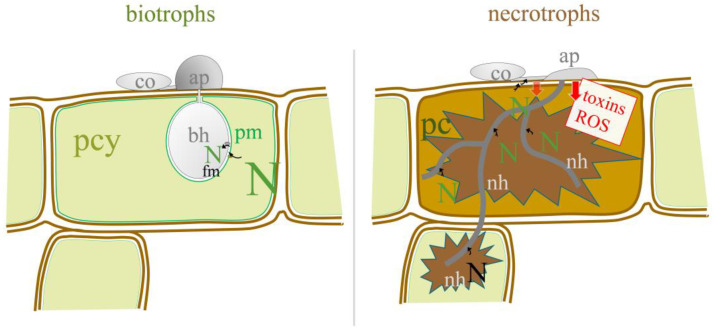
Model of nitrogen (N) availability in/on the host plant for biotrophic and necrotrophic fungi. co, conidia; ap, appressoria; bh, biotrophic hyphae; nh, necrotrophic hyphae; pcy, plant cytoplasm; pc, plant cell; pm, plasma membrane.

Since the plant surface is essentially devoid of nitrogen, fungi have developed lifestyle-specific strategies to access the nitrogen of their host plants. The pre-penetration infection structures of biotrophic and hemibiotrophic fungi develop in the absence of external nitrogen [15]. Therefore, biotrophs and hemibiotrophs critically depend on purine degradation and active urease, and urease inhibitors may therefore interfere with plant invasion by these pathogens [11]. In contrast, necrotrophic fungi secrete toxins and/or generate reactive oxygen species (ROS) before entering the plant [24] and killing host cells, and nitrogenous molecules may become available prior to invasion (Figure 1). Therefore, we tested five urease inhibitors for their ability to prevent or delay the disease progression of fungal pathogens differing in lifestyle.

### 3.1. Inhibition of Urease Activity by Two Natural and Three Novel Synthetic Compounds

**Figure 2:**
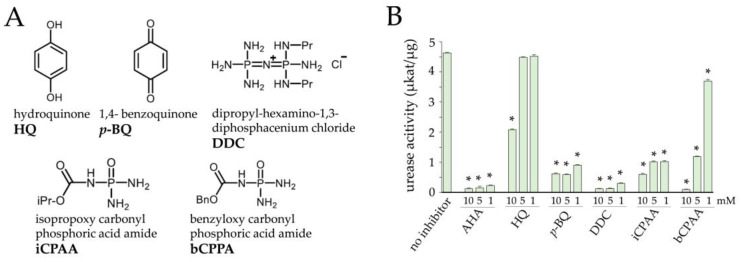
Chemical structure of the putative urease inhibitors (**A**) and urease activity in the presence of different concentrations of five putative urease inhibitors (**B**) Data are means of three independent experiments. Error bars are standard deviations, asterisks indicate statistically significant differences (Tukey’s post hoc test at *p* ≤ 0.05).

Urease inhibitors used in this study (Figure 2A) exhibit different modes of action [25]. p-BQ and HQ inhibit ureolysis by holding the flap in a configuration that sterically prevents urea from accessing the catalytic Ni center [26,27]. iCPAA, bCPAA, and DDC bind to the active site of the enzyme and constitutively inhibit urease activity [27]. Urease assays performed in the presence of these compounds indicated the highest inhibitory activity for p-BQ and iCPAA, followed by DDC, bCPAA, and AHA, which was used as a reference compound (Figure 2B). HQ showed significant inhibition at the highest concentration of 10 mM only (Figure 2B). p-BQ inhibited the activity of urease more efficiently than HQ, with very high inhibitory activity even at a concentration of 1 mM (Figure 2B, p-BQ).

### 3.2. Efficacy of Urease Inhibitors against the Obligate Biotrophs Blumeria graminis f. sp. tritici and Uromyces viciae-fabae

**Figure 3:**
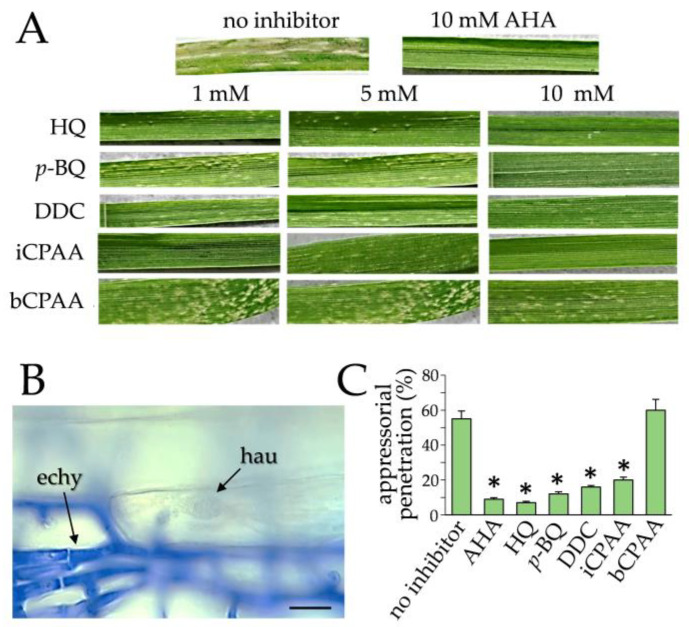
Effect of the urease inhibitors *p*-BQ, HQ, DDC, iCPAA, and bCPAA on disease development of *B. graminis* f. sp. *Tritici*. (**A**) Disease symptoms of *B. graminis* f. sp. *Tritici*-inoculated leaves pre-treated with different concentrations of urease inhibitors. Leaves without inhibitor (no inhibitor) and leaves treated with AHA served as controls. Photographs were taken at 12 dpi. (**B**) Microscopy of epicuticular hyphae (echy) and haustoria (hau) of *B. graminis* f. sp. *Tritici* on untreated wheat leaves. The leaf sample was stained with 0.1% aniline blue; epicuticular hyphae are strongly stained, haustoria remained unstained. (**C**) Quantification of appressorial penetration at 48 hpi, as indicated by the number of haustoria developed from 100 appressoria on untreated or inhibitor-treated leaves. All experiments were performed as three independent repeats. Error bars are standard deviations, asterisks indicate statistically significant differences (Tukey’s post hoc test; *p* ≤ 0.01).

At 12 days post-inoculation (dpi), dense powdery mildew pustules had formed on non-treated wheat leaves (Figure 3A, no inhibitor). At a concentration of 10 mM, all inhibitors except for bCPAA strongly inhibited mildew infection. Intriguingly, HQ and iCPAA yielded strong protection at all concentrations tested, whereas DDC and, to a lesser extent, p-BQ were moderately active (Figure 3A). Microscopy indicated that on untreated leaves, conidia had formed primary and appressorial germ tubes, from which appressoria developed. By 48 hpi, abundant epicuticular hyphae (Figure 3B; echy) had formed with differentiated appressoria, which had breached the epidermal cell wall and formed haustoria (Figure 3B; hau). On leaves treated with 10 mM p-BQ, HQ, DDC, and iCPAA, the formation of appressoria was only slightly reduced (data not shown), but plant invasion and differentiation of haustoria were significantly inhibited (Figure 3C). Consistent with previous data [11], AHA also inhibited appressorial penetration and haustorium formation and effectively protected wheat leaves from B. graminis f. sp. tritici infection. The data indicate that this biotrophic powdery mildew fungus requires a functional urease for appressorial host invasion and pathogenesis.

Importantly, the phytotoxic effects of the inhibitors used in this study were not observed, as on leaves treated with 10 mM concentrations, neither chloroses nor necroses occurred. As an example, inhibitor-treated maize leaves are shown (Supplemental Figure S1).

**Figure 4:**
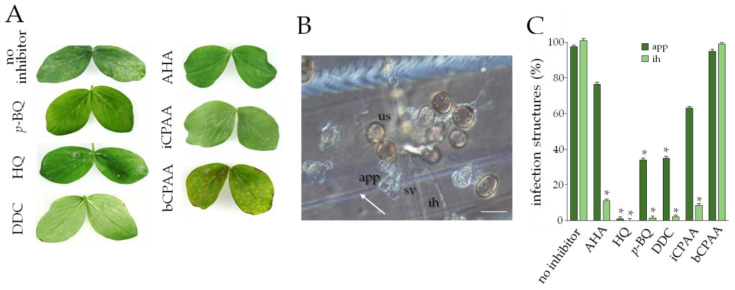
Effect of urease inhibitors on the broad bean rust fungus *U. viciae-fabae*. (**A**) Leaves were sprayed with urediniospore suspensions with or without 10 mM of the urease inhibitors *p*-BQ, HQ, DDC, iCPAA, or bCPAA, respectively, or with the reference compound AHA, and incubated. Leaves were photographed at 12 dpi. (**B**) Microscopy of infection structures on thigmo-inductive polyethylene sheets. us, urediniospore; app, appressorium; sv, substomal vesicle; ih, infection hypha. The arrow indicates a thigmo-inductive ridge. Size bar is 20 µm. (**C**) Quantification of appressoria and infection hyphae on thigmo-inductive polyethylene sheets in the absence (no inhibitor) or presence of 10 mM of the inhibitors. Infection structures were counted at 24 hpi. All experiments were performed as three independent repeats. Error bars are standard deviations, asterisks indicate statistically significant differences (Tukey’s post hoc test at *p* ≤ 0.01).

To investigate the effect of urease inhibitors on another biotrophic fungus, we employed the broad bean rust fungus U. viciae-fabae. Like most rust fungi, U. viciae-fabae deposits an appressorium on a stomatal pore [14] to invade the host leaf. Subsequently, an infection hypha and a haustorial mother cell are differentiated in the intercellular space, and a haustorium, surrounded by the plasma membrane of the plant, is formed after invading the host cell [15]. On broad bean leaves that had not been treated with a urease inhibitor, successful U. viciae-fabae infection was indicated by large numbers of uredosori at 12 dpi (Figure 4A, no inhibitor). In contrast, except for the urease inhibitor bCPAA, application of the other urease inhibitors at a concentration of 10 mM, including the reference inhibitor AHA, strongly reduced rust pustule formation by the end of the experiment at 12 dpi.

As rust hyphae developing in the intercellular space are difficult to visualize, polyethylene sheets with thigmo-inductive ridges [16] were inoculated with urediniospores in the presence or absence of the inhibitors. Untreated urediniospores germinated and, after contact with a ridge (Figure 4B, arrow), differentiated appressoria. Subsequently, substomatal vesicles and infection hyphae formed (Figure 4B). In the presence of the inhibitors p-BQ, HQ, DDC, and iCPAA, appressoria and infection hyphae were formed at reduced rates (Figure 4C), clearly indicating that a functional urease is required for infection-related morphogenesis of the broad bean rust fungus.

### 3.3. Effect of Urease Inhibitors on Infection of Maize Leaves by C. graminicola

**Figure 5:**
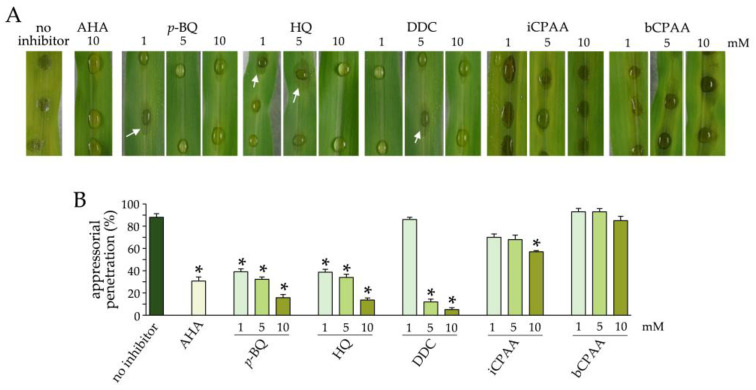
Maize leaf segments inoculated with conidial suspensions with or without urease inhibitors. Leaves were photographed at 3 dpi. Bars show rates of in planta-differentiated infection hyphae at 72 hpi. All assays were performed as three independent repeats. Error bars are standard deviations, and asterisks indicate statistically significant differences (Tukey’s post hoc test at *p* ≤ 0.01).

To investigate whether the urease inhibitors tested here would protect maize leaves from anthracnose disease, we added 1, 5, or 10 mM of each substance to the infection droplet. In the absence of urease inhibitors, the WT strain of C. graminicola caused significant disease symptoms at 3 dpi (Figure 5A, no inhibitor). Supplementing the infection droplets with the urease inhibitors AHA, p-BQ, HQ, or DCC strongly reduced disease severity, and anthracnose disease symptoms did not occur. In some cases, however, water-soaked lesions were observed (Figure 5A, arrows). The inhibitors iCPAA and bCPAA had no observable inhibitory activity against the maize anthracnose fungus (Figure 5A). Microscopy clearly allowed us to determine successful appressorial penetration (Figure S2). Quantification of infection structures showed that in the presence of AHA, p-BQ, HQ, or DCC, germination and appressorium differentiation were delayed, and appressorial penetration competence was reduced in a concentration-dependent manner, as indicated by quantification of in planta-differentiated infection hyphae (Figure 5B).

**Figure 6:**
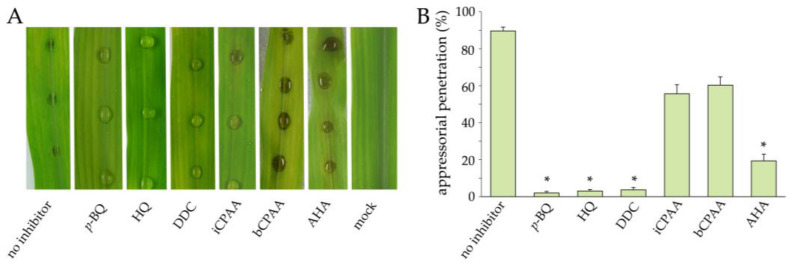
Maize leaf segments inoculated with conidial suspensions of *C. graminicola* 5 days after spraying with urease inhibitors (10 mM). (**A**). Inoculated leaves that had not been treated with an inhibitor (no inhibitor) served as positive controls. Mock-inoculated leaves (no fungus) served as negative controls; AHA served as a reference compound. Infected leaves were photographed at 3 dpi. (**B**). Rates of appressorial penetration at 72 hpi. Error bars are standard deviations, asterisks indicate statistically significant differences (Tukey’s post hoc test at *p* ≤ 0.01). Assays were performed as three independent repeats.

To test for the stability of the urease inhibitors on the leaf surface, we applied the compounds by spraying the entire leaf surface with 10 mM concentrations to the point of run-off, and droplet-inoculated the leaves five days after this treatment with conidial suspensions of C. graminicola. Disease symptoms were analyzed macroscopically and appressorial penetration rates were quantified microscopically at 3 dpi (Figure 6). Maize leaves pre-treated with p-BQ, HQ, DDC, and AHA did not show anthracnose symptoms, suggesting a stable protective effect of these inhibitors over at least five days (Figure 6A). The lack of disease symptoms correlated with the reduction in appressorial penetration rates on leaves pre-treated with these chemistries (Figure 6B). In comparison, iCPAA and bCPAA had no obvious protective effect (Figure 6A), similar to the results of the experiments in which the inhibitors were directly added to the infection droplet (compared with Figure 5). Failure to inhibit the occurrence of anthracnose disease symptoms by iCPAA and bCPAA agrees with the minor and not statistically significant reduction in penetration rates, as compared with the no-inhibitor control (Figure 6B).

### 3.4. Effect of Urease Inhibitors on Necrotrophic Plant Pathogens

**Figure 7:**
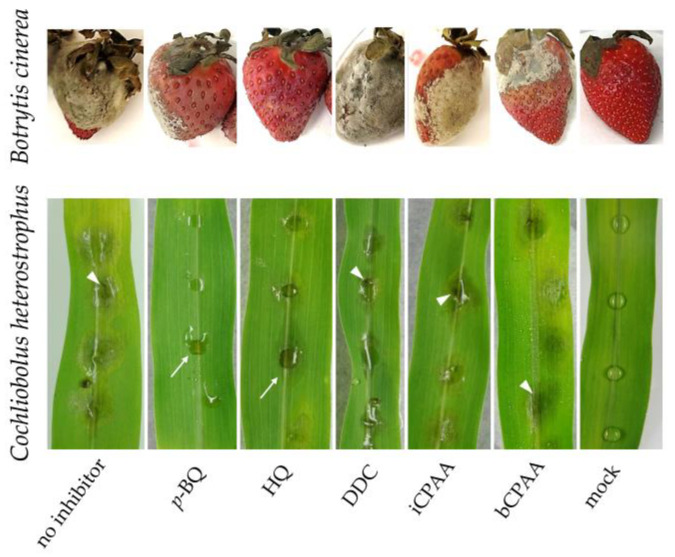
Effect of urease inhibitors on the necrotrophs *B. cinerea* and *C. heterostrophus*. Strawberry fruits were spray-inoculated with compound-containing spore suspensions and photographed at 72 hai. Disease symptoms caused by *C. heterostrophus* were photographed at 48 pi. All inhibitors were applied at a concentration of 10 mM; mock-treated plants and untreated inoculated plants served as controls. Arrows indicate water-soaked lesions, arrowheads indicate necrotic tissue.

The effect of urease inhibition on fungi with a necrotrophic lifestyle, i.e., the Southern Leaf Spot fungus C. heterostrophus and the grey mold fungus B. cinerea [28], were tested using 10 mM each of p-BQ, HQ, DDC, iCPAA, or bCPAA. B. cinerea has a broad host range of more than 1000 plant species, including commercially important fruits such as strawberries, resulting in severe yield and quality losses [29]. On non-treated plants, clear disease symptoms were observed at 72 hpi. Interestingly, HQ and p-BQ had some protective effect over 72 hpi (Figure 7), but fruits developed typical soft-rot symptoms thereafter (data not shown). In the presence of the other inhibitors, the occurrence of disease symptoms was not delayed, and was clearly visible even at 72 hpi (Figure 7; B. cinerea).

p-BQ also reduced the disease symptoms caused by C. heterostrophus on maize leaf segments, with mild water-soaked lesions visible at 48 hpi (Figure 7; p-BQ, arrow). In comparison, in the presence of HQ, the fungus developed faster, as indicated by more strongly enlarged lesions (Figure 7; HQ, arrow). In the presence of DDC, iCPAA, or bCPAA, C. heterostrophus had formed severe lesions, with necroses at the infection center (Figure 7; DDC, iCPAA, bCPAA, arrowheads). However, none of the urease inhibitors tested reduced the development of disease symptoms caused by necrotrophs effectively and durably. Microscopy revealed that in fruit or leaf tissue with minor symptoms, hyphae were massively present (data not shown), which further indicated that urease inhibitors did not effectively inhibit fungal ingress.

## 4. Discussion

Plant pathogenic fungi exhibiting a biotrophic or hemibiotrophic lifestyle gain access to the nitrogen of their host plants only after establishing a compatible parasitic interaction [30,31]. Appressoria differentiated on the plant surface by *Colletotrichum* or *Magnaporthe* species are spatially separated from the nutrient resources of the host [32]. Therefore, the purine degradation pathway is thought to be required for providing ammonium for the substantial pathogenesis-related reprogramming of RNA and protein biosynthesis prior to and at plant invasion [33]. Implicitly, inhibitors of urease, the last enzyme of the purine degradation pathway, could represent novel effective fungicidal chemistries, allowing them to control diseases caused by pathogens with biotrophic and hemibiotrophic lifestyles. However, as previously only AHA, a known competitive inhibitor of urease, has been analyzed, we decided to increase the spectrum of examined inhibitors. Moreover, as the role of urease in pathogenesis is unknown in necrotrophs, we expanded the spectrum of fungi by including two classical necrotrophs, i.e., the polyphagous grey rot fungus *B. cinerea* and the maize pathogen *C. heterostrophus*. The putative urease inhibitors used in this study had previously been identified as compounds affecting urea transformation in soils. We took advantage of these in vitro inhibitor studies to test the disease-protecting activities of five different inhibitors belonging to different chemical classes. Using commercially available jackbean urease, the urease inhibitory activity of the chemistries used was demonstrated, three of which were described as urease inhibitors for the first time in the present study. AHA, which like DDC was a strong inhibitor of jackbean urease activity in vitro, protected plants from infection by all the biotrophs and the hemibiotrophs tested here. Interestingly, the phosphoric acid amides, iCPAA and bCPAA, effectively inhibited jackbean urease at 10 mM concentrations in vitro, but only iCPAA interfered with wheat powdery mildew and broad bean rust infection. Neither iCPAA nor bCPAA affected the hemibiotroph *C. graminicola* nor were these chemistries effective against the necrotrophs tested. Reversely, HQ was moderately inhibitory in the urease activity assay only at the highest concentration tested but like *p*-BQ, effectively inhibited mildew, rust, and *C. graminicola* infections. These apparent discrepancies between inhibitory effects on purified jack bean urease and activity against the ureolytic bacterium *Klebsiella pneumoniae* have also been observed in a large screen comparing 71 commercially available urease inhibitors [18]. It is very likely that the discrepancies between in vitro urease activity assays and control of biotrophic and hemibiotrophic plant pathogenic fungi are due to the differential cell wall and/or membrane permeability of the compounds tested. However, the fact that biotrophs and hemibiotrophs are more susceptible to several of the inhibitors tested than necrotrophs strengthens the conclusion of Perino and co-workers that the biotrophic and hemibiotrophic lifestyle depends on the generation of ammonium via the purine degradation pathway, which in turn underlines the role of urease in pathogenesis [11]. The results are consistent with the assumption that nitrogen is required for metabolic re-programming in the invasive phase of infection. Interestingly, the previously tested urease inhibitor AHA was not effective in preventing infection caused by both *C. heterostrophus* and *B. cinerea*, leading to the hypothesis that, in contrast to biotrophic and hemibiotrophic fungal pathogens, necrotrophs gain immediate access to the host nitrogenous compounds by killing host cells prior to invasion. Like AHA, neither DDC nor iCPAA or bCPAA were able to inhibit infection by these pathogens. Symptoms caused by *B. cinerea* and *C. heterostrophus*, although to different extents, appeared to be slightly reduced by HQ and *p*-BQ. Despite the reduced severity of disease symptoms at the early stages of pathogenesis, microscopy revealed that these necrotrophic fungi had invaded the host tissues, and severe symptoms occurred with some delay. The differences in the efficacies of quinone-based compounds and the other inhibitors tested may be due to the peculiar mode of action of *p*-BQ and HQ [18]. These compounds represent redox systems and may act as ROS-scavenging chemistries [18]. In planta-developing pathogenic hyphae of necrotrophic fungi such as *Botrytis* spp. generate ROS, which kill host cells and cause major tissue damage well in advance of the growing hyphae. The protective effect of radical scavengers, such as quinone-based chemistries against ROS, has been extensively discussed and reviewed elsewhere [34,35,36], and the delayed infection of *p*-BQ- and HQ-treated maize leaves and strawberry fruits by necrotrophs may indeed be due to the ROS-scavenging effect of these compounds.

## 5. Conclusions

Overall, the role of urease in the early stages of infection, as evidenced by previous gene deletion studies [11], was confirmed through chemical inhibition of urease activity in hemibiotrophic and biotrophic pathogens infecting monocot and dicot plants. Treatment of economically relevant pathogens such as the maize anthracnose fungus *C. graminicola*, the powdery mildew fungus *B. graminis* f. sp. *tritici*, and the rust fungus *U. viciae-fabae* with four out of five different inhibitors belonging to different chemical classes has been shown to reduce disease symptoms. In comparison, the inhibitors only slightly affected infections by the necrotrophic pathogens *B. cinerea* and *C. heterostrophus*, demonstrating that the inhibitory effect strongly depends on the fungal lifestyle.

The discovery of new fungicide targets has significant relevance as more and more active ingredients are banned, resulting in reduced numbers of pesticides exhibiting distinct modes of action. It is widely accepted that reducing the range of registered fungicides may lead to the rapid development of resistance to the few remaining fungicides. It is also accepted that chemistries with three distinct modes of action are needed to avoid the development of fungicide resistance [37]. Importantly, in 1973, for only 510 out of 5577 interactions between crops and pathogens, pests, and weeds, fewer than three chemistries with distinct modes of action were available, which is less than 10%. In 2018, however, this value had increased to 3539 interactions, i.e., to 63.5% of the interactions [37]. The banning of plant-protecting chemistries in agriculture strongly argues that the development of novel fungicides with new modes of action is urgently needed to maintain an effective arsenal of chemistries to control devastating diseases. Along this line of argument, specific urease inhibitors, as characterized in this study, might add to the complexity of fungicidal compounds and contribute to plant health.

## Data Availability

Data is contained within the article and supplementary material.

## Supplementary Materials

The following supporting information can be downloaded at: https://www.mdpi.com/article/10.3390/jof9070708/s1, Figure S1: Analysis of phytotoxicity of urease inhibitors using spray application of 10 mM solutions of *p*-BQ, HQ, DDC, iCPAA, and bCPAA, respectively. Photographs were taken at 5 dpt. Figure S2: Microscopy of *C. graminicola* on detached maize leaves at 48 hpi. Maize leaves were inoculated with the WT strain CgM2. Ten µl of a conidial suspension containing 10^6^spores/ml and 0.02% (*v*/*v*) Tween 20, with or without 10 mM of the urease inhibitor AHA were inoculated onto the intact leaf surface. co, conidia; app, appressoria; ih, infection hyphae. Prior to microscopy, 0.01% (*v*/*v*) Aniline blue was applied to leaves to discriminate structures formed on the plant surface (co; app, in blue) from those formed in the plant (ih). Size bar = 20 µm.
