# Supplementary material for: Natural Urease Inhibitors Reduce the Severity of Disease Symptoms, Dependent on the Lifestyle of the Pathogens
Source: J Fungi (Basel). 2023 Jun 28;9(7):708. doi: 10.3390/jof9070708 (PMC10381680; doi:10.3390/jof9070708)
Supplement: Supplementary file 1 [file jof-09-00708-s001.zip › jof-2468549-supplementary.pdf]

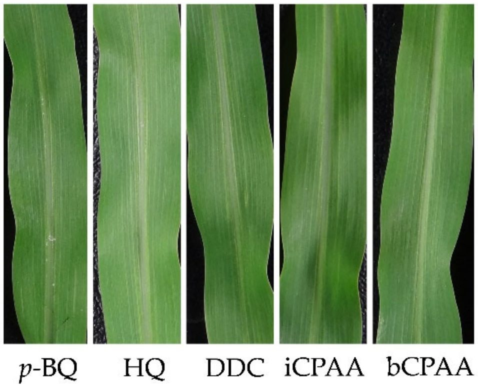

Figure S1. Analysis of phytotoxicity of urease inhibitors using spray application of 10 mM solutions of *p*-BQ, HQ, DDC, iCPAA, and bCPAA, respectively. Photographs were taken at 5 dpt.

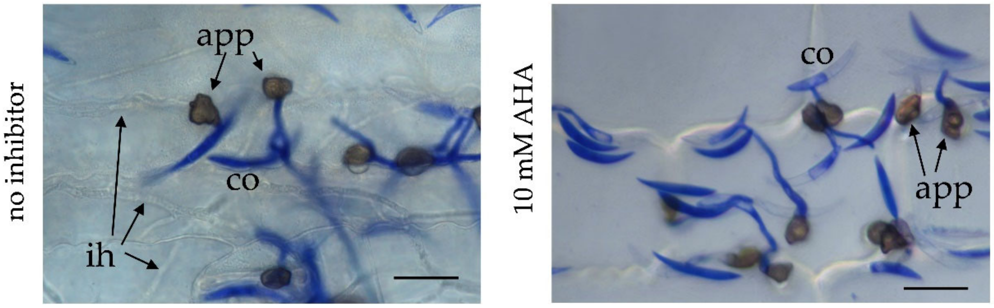

Figure S2. Microscopy of *C. graminicola* on detached maize leaves at 48 hpi. Maize leaves were inoculated with the WT strain CgM2. Ten µl of a conidial suspension containing 10^6spores/ml and 0.02% (v/v) Tween 20, with or without 10 mM of the urease inhibitor AHA were inoculated onto the intact leaf surface. co, conidia; app, appressoria; ih, infection hyphae. Prior to microscopy, 0.01% (v/v) Aniline blue was applied to leaves to discriminate structures formed on the plant surface (co; app, in blue) from those formed in the plant (ih). Size bar = 20 µm.
